# PREDICTORS OF DEMENTIA IN OLDER ADULTS: VARIATION BY NATIVITY STATUS AND RACE-ETHNICITY

**DOI:** 10.1093/geroni/igz038.1736

**Published:** 2019-11-08

**Authors:** Heehyul Moon, Adrian Badana, So-Yeon Hwang, Jeanelle Sears, William E Haley

**Affiliations:** 1 University of Louisville, Louisville, Kentucky, United States; 2 School of Aging Studies, University of South Florida, Tampa, Florida, United States; 3 NONE, Cincinnati, Ohio, United States; 4 University of South Florida, Tampa, Florida, United States

## Abstract

Among many possible factors that are associated with increased risk for dementia, one important topic is the role of race and ethnicity. However, previous studies did not examine rates separately by racial/ethnic groups and by nativity status, and little of this research comes from nationally representative data. The current study investigated different predictors of dementia by race/ethnicity and nativity status using Round 1 of the National Health and Aging Trends Study (NHATS, N=7,473). Our stratified log-binominal regression analyses by race/ethnicity (Non-Hispanic White (NHW), non-Hispanic Black (NHB), Hispanic, and Other) show that the relationship of nativity status to dementia (Probable dementia vs Possible and no dementia) is likely determined by multiple factors (e.g., education, # of cardiovascular conditions/risk factors, # in household) that may vary across race and ethnicity. The relative risks of Probable dementia were higher among immigrants in the Hispanic, and other groups, but lower among immigrants in NHBs, although the relative risk of dementia among NHBs was marginally significant. Gender was not a significant factor of Probable dementia across the four groups after adjustment for other variables including nativity status. Older age was significantly associated with the higher prevalence of Probable dementia across the four groups. However, other risk factors for dementia varied considerably between the four racial/ethnic groups after controlling for nativity status. Healthcare providers systems must be prepared to deliver culturally competent supports and services to diverse groups of older Americans and their family members.

